# Multiple mediating roles of physical and mental health in the effects of physical exercise on prosocial behavior in junior high school students

**DOI:** 10.3389/fpsyg.2025.1605442

**Published:** 2025-07-10

**Authors:** Liangru Guo, Liang Jiang, Huizhi Huang

**Affiliations:** School of Sports Science, Hengyang Normal University, Hengyang, China

**Keywords:** physical exercise, junior high school students, prosocial behavior, physical and mental health, adolescents

## Abstract

**Objective:**

To examine the associations between physical exercise and prosocial behavior among junior high school students, and to explore the potential mediating roles of physical and mental health in these associations, providing insights for interventions targeting prosocial behavior.

**Methods:**

Using data from the China Education Panel Survey 2014–2015, we analyzed 7,605 junior high school students’ self-reported measures of prosocial behavior, physical exercise, and physical and mental health. An OLS regression model was employed to assess associations, and a multiple mediation model (PROCESS Model 4 with 5,000 bootstraps) was used to test indirect effects.

**Results:**

Physical exercise was positively associated with prosocial behavior (*β* = 0.191, *p* < 0.001), with stronger associations observed among students with highly educated parents (*β* = 0.184, *p* < 0.001) and male students (*β* = 0.178, *p* < 0.001). Both physical health [*β* = 0.018, 95% CI (0.012, 0.024), *p* < 0.001] and mental health [*β* = 0.009, 95% CI (0.005, 0.013), *p* < 0.001] showed significant indirect associations in the link between physical exercise and prosocial behavior, with physical health accounting for over twice the indirect association of mental health.

**Conclusion:**

Physical and mental health may play mediating roles in the relationship between physical exercise and prosocial behavior among junior high school students. Schools and families should encourage students’ participation in physical exercise, which may support their physical and mental well-being and foster prosocial behavior.

## 1 Introduction

Prosocial behavior refers to behavior that is beneficial to others and conducive to promoting positive social relationships, including friendly behavior, helpful behavior, and adherence to social norms (Pfattheicher et al., 2022). Adolescents are in a critical period of social development, including the development of prosocial behavior (Eisenberg and Koller, 2001). It has been demonstrated that prosocial behavior promotes academic achievements (Gerbino et al., 2018), enhances the sense of well-being (Nelson et al., 2016), improves emotional and social cognitive abilities (Metzger et al., 2018), and helps better cope with anxiety and depression caused by social competition, confusion, and other negative emotions (Storch and Masia-Warner, 2004).

Exercise is planned, structured, and repetitive bodily movement aimed at improving or maintaining one or more components of physical fitness (Dasso, 2019). Physical exercise has been shown to promote the development of prosocial behavior during adolescence (Konukman et al., 2022), which is largely a result of improving self-efficacy and the ability to deal with negative emotions through the development of social personality traits. Moreover, adolescents who regularly participate in sports activities have significantly lower levels of interpersonal anxiety and significantly higher levels of self-resilience and prosocial behavior (Kavussanu and Al-Yaaribi, 2019; Wan et al., 2021).

Based on Bandura’s social cognitive theory, adolescence is a critical stage for social comparison and identity formation, and adolescents’ mental health (e.g., emotional states such as depression and loneliness) may play a mediating role in the relationship between their physical activity and prosocial behavior (Bandura, 2001). Physical activity can enhance adolescents’ social adjustment by boosting self-efficacy and facilitating observational learning, which can reduce negative social cognitions and improve psychological well-being (Hawkley and Cacioppo, 2010). Meanwhile, according to self-determination theory, physical activity that satisfies adolescents’ autonomy, sense of competence, and relational needs will further alleviate emotional problems and stimulate benign social cognitions, which in turn will promote prosocial behavior. Concurrently, embodied cognition theory provides complementary explanatory power regarding the mediating role of subjective physical health. This theoretical framework posits that improvements in physical coordination, fitness levels, and bodily awareness may contribute to improved non-verbal communication and body confidence, thereby facilitating more positive social interactions. Together, these theoretical perspectives—social cognitive theory addressing mental health pathways and embodied cognition theory explaining physical health mechanisms—offer an integrated framework for understanding how both psychological and physiological factors mediate the effects of physical activity on adolescents’ prosocial development.

**Figure 1:**
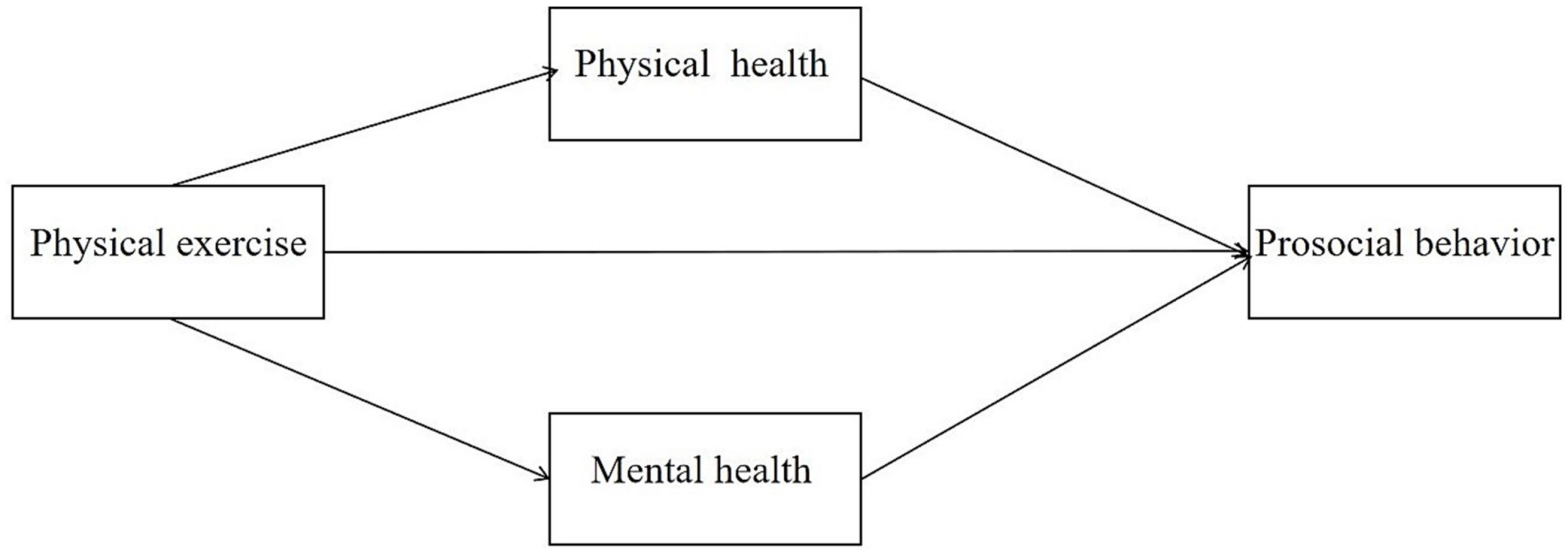
Hypothetical model of multiple mediation of physical and mental health in the relationship between physical exercise and prosocial behavior.

Whereas the effects on prosocial behavior of adolescents are not instantaneous, most studies focusing on mental health mediators, such as sports learning motivation (Hui et al., 2022), self-perception (Konukman et al., 2022), subjective well-being (Duan et al., 2022), and basic psychological needs (Hodge and Gucciardi, 2015), have demonstrated effects on prosocial behavior. A few studies have centered on the impact of physical health mediators such as subjective health level (Perc et al., 2012), and health-related quality of life (Peacock, 2020). Building on these theoretical foundations and empirical findings, we propose three hypotheses: Hypothesis 1: Physical exercise is positively associated with adolescents’ prosocial behavior. Hypothesis 2: Physical exercise is linked to adolescents’ prosocial behavior through enhanced physical health. Hypothesis 3: Physical exercise is linked to adolescents’ prosocial behavior through improved mental health. This study makes three key contributions: (1) providing the first large-scale national evidence from China (using CEPS 2014–2015 data) to examine how physical exercise may influence adolescents’ prosocial behavior through physical and mental health; (2) systematically comparing the potential mediating roles of physical health versus mental health to determine their relative importance in this relationship; and (3) investigating whether these relationships vary across key subgroups such as gender and parental education levels. By incorporating these elements into a comprehensive multiple mediation framework, this study seeks to advance theoretical understanding of the mechanisms linking physical exercise to prosocial behavior while offering evidence-based insights for targeted interventions during adolescence, a crucial developmental stage. Figure 1 depicts the proposed hypothetical model.

## 2 Materials and methods

### 2.1 Data sources

The China Education Panel Survey (CEPS), designed and implemented by the National Survey Research Center (NSRC) at Renmin University of China, is a large-scale nationally representative panel survey. This study utilizes secondary data from CEPS, for which written informed consent has been obtained from all participants (or their guardians) during the original data collection. The data presented in this study are available at http://ceps.Ruc.edu.cn/. Since the launch of the first phase of the survey in July 2013, about 20,000 adolescents from 438 classes in 112 schools, as well as their parents, teachers, and school leaders, have been selected as the survey samples in 28 counties across the country using a multilevel and multistage sampling method. The CEPS data follow the principles of probability sampling that is hierarchical and multistage, with probability proportional to size (PPS), and its samples are representative of the whole country. This study used data from the 2014–2015 panel survey, which covers students’ physical exercise, prosocial behavior, and many variables that predict prosocial behavior, such as the nature of the family and school. Our study consisted of a rigorous two-stage screening process conducted independently by two researchers, with differences verified by both researchers. Eligibility required: (1) successful follow-up in both 2013–2014 and 2014–2015 waves; (2) complete data on individual characteristics (age, gender, grade), family characteristics (parental education, household income), and school characteristics (school type, location). From the initial 10,750 observations, first-stage exclusions removed 830 unsuccessful follow-ups and 471 newly added 2014–2015 samples. Second-stage screening excluded 1,844 cases with missing values: 9 exceeding daily exercise limits (>360 min), 66 lacking prosocial behavior measures, and 1,769 with incomplete covariates. The final sample size was 7,605.

### 2.2 Model construction

In order to examine the effects and mechanism of physical exercise on adolescents’ prosocial behavior, this paper constructed an OLS regression model Equation 1 as follows:


(1) $$Y_{i}=\beta_{0}+\beta_{1}X_{i}+\beta_{2}M1_{i}+\beta_{3}M2_{i}+\beta_{4}{\mathrm{COV}}_{i}+\varepsilon_{i}$$

Where, $Y_{i}$ is the dependent variable, representing the prosocial behavior of individual *i,* $X_{i}$ is the independent variable, representing that individual *i* is physically active; $M1_{i}$ represents the physical health status of individual *i*; $M2_{i}$ represents the mental health status of individual *i*; ${\mathrm{COV}}_{i}$ represents a series of control variables for sample *i*, including variables at the individual, family, and school levels; $\varepsilon_{i}$ represents the random perturbation term; $\beta_{0}$ represents the constant term; $\beta_{1}$ is the coefficient of regression corresponding to the independent variable $X_{i}$ ; $\;\beta_{2}$ and $\beta_{3}$ are the estimated coefficients corresponding to $M1_{i}$ and $M2_{i}$ ; $\beta_{4}$ is the regression coefficients corresponding to each control variable.

In addition, in order to identify the mediating role of physical health status and mental health status in the effects of physical exercise on adolescents’ prosocial behavior, this paper draws on the test of multiple mediating effects proposed by Hayes (2009) by constructing the following model and testing it in conjunction with the baseline model (1).


(2) $$Y_{i}=a_{0}+a_{1}X_{i}+{\text{λCOV}}_{i}+\mu_{i}\;$$

(3) $$M1_{i}=b_{0}+b_{1}X_{i}+{\text{ΩCOV}}_{i}+v_{i}\;$$

(4) $$M2_{i}=c_{0}+c_{1}X_{i}+{\text{pCOV}}_{i}+u_{i}$$

The variables in Equations 2–4 are the same as in the baseline model. $a_{0}$ , $b_{0}$ and $c_{0}$ are the constant terms of the three models, respectively. $a_{1}$ , $b_{1}$ and $c_{1}$ are the total effects of physical exercise on adolescents’ prosocial behavior, the effects of physical exercise on the mediating variable, physical health, and the effects of physical exercise on the mediating variable, mental health, and λ , Ω and p are the regression coefficients corresponding to each of the control variables of the three models. $\mu_{i}$ , $v_{i}$ and $u_{i}$ are the random perturbation terms of the three models. $\beta_{1}$ in model (1) represents the direct effect of physical exercise on adolescents’ prosocial behavior, $b_{1}\beta_{2}$ represents the mediating effect of physical health in physical exercise’s effects on adolescents’ prosocial behavior, and $c_{1}\beta_{3}$ represents the mediating effect of mental health in physical exercise’s effects on adolescents’ prosocial behavior. Therefore, $b_{1}\beta_{2}$ + $c_{1}\beta_{3}$ is the total mediating effect, and the total effect, direct effect and mediating effect satisfy the following relationship: $a_{1}$ = $\beta_{1}$ + $b_{1}\beta_{2}$ + $c_{1}\beta_{3}$ .

### 2.3 Variable setting

#### 2.3.1 Dependent variable

In this study, prosocial behavior was used as the dependent variable, which was measured by three specific items in the “Have you done any of the following behaviors in the past year?” section of the CEPS questionnaire. Three specific items in the section “Helping the elderly to do things,” “Being orderly and queuing up,” and “Being sincere and friendly to others” were used to measure prosocial behavior. Five options were set for each entry, “never, occasionally, sometimes, often, and always,” which were assigned values from 1 to 5. By summing up the scores of the three entries, a continuous variable ranging from 3 to 15 was constructed, with higher scores indicating more prominent prosocial behaviors. Cronbach’s alpha was 0.671 in this study.

#### 2.3.2 Independent variables

The independent variable was physical exercise time, which was measured based on the CEPS data on the frequency of exercise in a week and the length of a single exercise session. The sample extremes that answered “more than 360 min of exercise per session” (Hu and Yu, 2019) were excluded, and then the average daily exercise duration of adolescents was calculated according to the formula (average daily exercise duration = number of days of exercise per week × duration of exercise per day/7). In order to avoid the loss of zero-valued samples and to smooth the data distribution, the average daily exercise hours were adjusted by a constant term of 0.01 and then processed by taking the natural logarithm, and finally a continuous variable with an approximate normal distribution was obtained.

#### 2.3.3 Mediating variables

(1) Physical health, a subjective indicator, i.e., physical health awareness, was chosen to measure “How is your overall health now?” To measure it, a five-point Likert scale was used, assigning values from 1 to 5 to “very bad, not so good, fair, relatively good, and very good” respectively (Hongchang et al., 2025).

(2) Mental health, which mainly investigates the emotional and mental state in the past seven days, consists of 10 entries: “depressed, unable to concentrate on tasks, unhappy, feeling that life is pointless, lacking motivation, sad, nervous, excessively worrying, having a premonition that something bad is going to happen, overly energetic/having difficulty in focusing in class,” and the options are “very bad, not good, fair, relatively good, very good.” In this paper, each entry is assigned a value in reverse and then summed up to get the mental health status variable, which has a value range of 10 to 50. Cronbach’s alpha was 0.912 in this study.

#### 2.3.4 Control variables

The CEPS “Student Questionnaire,” “Parent Questionnaire” and “Principal Questionnaire” were subjected to the following control variables: gender, only child status, boarding, family economic status, household registration type, parents’ highest educational attainment, parents’ educational expectations, parental relationship, school nature, school location, and school sports facilities (Table 1).

**Table 1:** Variable assignment and descriptive statistics.

Variable	Variable definition	Sample size	Mean	Standard deviation	Minimum value	Maximum value
Dependent variable						
Prosocial behavior	Continuous variables (3–15)	7,605	11.40	2.265	3	15
Independent variable						
Physical exercise time	Natural logarithm of average daily physical exercise time	7,605	2.53	1.510	-5	6
Control variable						
Gender	1 = male, 0 = female	7,605	0.52	0.500	0	1
Only child status	1 = yes, 0 = no	7,605	0.44	0.497	0	1
Boarding	1 = yes, 0 = no	7,605	0.31	0.463	0	1
Family economic status	1 = very difficult, 2 = relatively difficult, 3 = moderate, 4 = relatively wealthy, 5 = very wealthy	7,605	2.95	0.608	1	5
Household registration type	1 = rural, 0 = non-rural	7,605	0.53	0.499	0	1
Parents’ highest education attainment	1 = bachelor’s degree and above, 0 = bachelor’s degree or below	7,605	0.13	0.333	0	1
Parents’ educational expectations	1 = bachelor’s degree and above, 0 = bachelor’s degree or below	7,605	0.65	0.477	0	1
Parental relationship	1 = good, 0 = bad	7,605	0.90	0.303	0	1
School nature	1 = public, 0 = private	7,605	0.93	0.247	0	1
School location	1 = rural, 0 = non-rural	7,605	0.20	0.399	0	1
Sports facilities	Continuous variables (3–9)	7,605	4.94	1.218	3	9
Mediating variable						
Physical health	1 = very bad, 2 = not so good, 3 = fair, 4 = relatively good, 5 = very good	7,605	3.87	0.93	1	5
Mental health	Continuous variable (10–50)	7,605	38.23	8.087	10	50

### 2.4 Statistical analysis

Data were organized and statistically analyzed using SPSS 21.0, and the effects of physical exercise on prosocial behavior were tested by OLS regression, using Model 4 and 5,000 Bootstraps in the PROCESS program for multiple mediation modeling.

To ensure the reliability and generalizability of our findings, we conducted robustness checks and heterogeneity tests in addition to the baseline regression analysis. Below, we detail the methodological approaches, justifications, and procedures for these analyses.

#### 2.4.1 Robustness checks

(a) Justification: Robustness checks are critical for verifying whether the baseline results are sensitive to alternative model specifications or measurement approaches. Given that self-reported measures of prosocial behavior may be subject to response bias, we employed a replacement variable method to mitigate potential measurement errors.

(b) Procedural Steps: Baseline Measure: The primary dependent variable was the student’s self-reported response to the question: “In the past year, were you able to help the elderly do things, follow order, queue up consciously, and treat people sincerely and kindly?”

Alternative Measure: To assess robustness, we replaced the dependent variable with the parent’s response to the parallel question: “In the past year, was your child able to help the elderly do things, follow order, queue up consciously, and treat people sincerely and kindly?” The response scale remained identical to ensure comparability.

Model Specification: We re-estimated the baseline regression using the alternative dependent variable while maintaining the same control variables and mediator inclusion sequence (physical health, mental health, and both).

(c) Reference Standards: Consistency in coefficient significance and direction across alternative specifications was used to evaluate robustness. Results were deemed robust when the key estimates remained statistically significant (*p* < 0.01) and aligned with baseline findings.

#### 2.4.2 Heterogeneity tests

(a) Justification: Prior research (Duan et al., 2022) suggests that the effects of physical exercise on prosocial behavior may vary by individual and familial characteristics. Heterogeneity tests were conducted to explore subgroup differences and enhance policy relevance.

(b) Procedural Steps: Subgroup Stratification: We stratified the samples by gender (male vs. female), and parental education level (high vs. low, defined by median split).

Model Re-estimation: The baseline regression model was separately estimated for each subgroup to compare coefficient magnitudes and significance levels.

(c) Reference Standards:

Group differences were assessed via coefficient comparisons. A stronger effect was inferred when one subgroup’s key coefficient was larger in magnitude and statistically significant (*p* < 0.01).

## 3 Results

### 3.1 Ordinary least squares (OLS) estimation

Table 2 shows the estimated results of the effects of physical exercise on adolescents’ prosocial behavior based on the OLS model. In Model 1, only the key core variable physical exercise is introduced to examine the correlation between the independent variables and the dependent variable; Models 2, 3, and 4 represent the estimation results of sequentially adding different control variables for individual, familial, and school characteristics; and Models 5, 6, and 7 represent the estimation results of sequentially adding the mediating variables of physical health and mental health, as well as both, on the basis of Model 3 (see Table 2).

**Table 2:** Effect of physical exercise on prosocial behavior of adolescents (OLS).

Variables	Model 1	Model 2	Model 3	Model 4	Model 5	Model 6	Model 7
Physical exercise	0.221 (19.721)***	0.217 (19.457)***	0.192 (17.307)***	0.191 (17.156)***	0.177 (15.99)***	0.183 (16.526)***	0.173 (15.688)***
Gender^a^	—	−0.122 (−11.017)***	−0.103 (−9.348)***	−0.102 (−9.195)***	−0.109 (−9.963)***	−0.105 (−9.581)***	−0.111 (−10.142)***
Only child status^b^	—	0.068 (5.842)***	0.03 (2.464)*	0.03 (2.411)*	0.026 (2.156)*	0.026 (2.127)*	0.024 (1.981)*
Boarding^c^	—	−0.011 (−0.908)	0.017 (1.368)	0.03 (2.336)*	0.031 (2.434)*	0.033 (2.534)*	0.033 (2.568)*
Family economic status	—	—	0.033 (2.895)**	0.032 (2.791)**	0.013 (1.126)	0.024 (2.048)*	0.009 (0.813)
Household registration type^d^	—	—	−0.028 (−2.132)*	−0.023 (−1.758)	−0.021 (−1.63)	−0.023 (−1.788)	−0.022 (−1.671)
Parents’ highest education attainment^e^	—	—	0.039 (3.27)**	0.037 (3.101)**	0.037 (3.138)**	0.037 (3.094)**	0.037 (3.127)**
Parents’ expectations^f^	—	—	0.136 (12.013)***	0.134 (11.811)***	0.131 (11.581)***	0.129 (11.357)***	0.127 (11.274)***
Parental relationship^g^	—	—	0.053 (4.846)***	0.053 (4.839)***	0.039 (3.566)***	0.038 (3.459)***	0.03 (2.725)**
School nature^h^	—	—	—	0.022 (1.877)	0.02 (1.746)	0.017 (1.436)	0.017 (1.436)
School location^i^	—	—	—	−0.044 (−3.543)***	−0.046 (−3.753)***	−0.046 (−3.717)***	−0.047 (−3.853)***
School sports facilities	—	—	—	−0.024 (−2.113)*	−0.023 (−2.047)*	−0.024 (−2.161)*	−0.023 (−2.092)*
Physical health	—	—	—	—	0.14 (12.624)***	—	0.12 (10.525)***
Mental health	—	—	—	—	—	0.111 (10.036)***	0.082 (7.245)***
Sample size	7,605	7,605	7,605	7,605	7,605	7,605	7,605
Constant term	10.563 (0.049)***	10.742 (0.063)***	9.71 (0.157)***	9.783 (0.206)***	8.863 (0.216)***	8.899 (0.223)***	8.341 (0.227)***
*R* ^2^	0.049	0.068	0.096	0.098	0.116	0.11	0.122
Adjusted *R*^2^	0.049	0.067	0.095	0.096	0.115	0.108	0.121
Amount of change in *R*^2^	0.049	0.019	0.028	0.002	0.019	0.012	0.013
*F*	388.93***	138.417***	89.5***	68.563***	76.869***	71.868***	75.612***

Reference group.

a male.

b Only child.

c Boarding.

d Rural.

e Bachelor’s degree and above.

f Parents’ high expectations.

g Good parental relationship.

h Public school.

i Rural.

***p < 0.001, **p < 0.01, *p < 0.05; standard errors in parentheses.

According to the regression results, the regression coefficients and significance of physical exercise and other control variables did not show significant changes, and the model fit results showed that the model is well set up, has good stability and strong explanatory power, and is thus suitable for causality analysis. In the process of constantly adding variables, the *R*^2^ value showed an upward trend, proving that the selection of control variables and mediating variables is reasonable and necessary. Among the seven models, the estimated coefficients of physical exercise passed the 1% significance check, and the signs were all positive, indicating that the physical exercise time has a positive effect on adolescents’ prosocial behavior, and Hypothesis 1 of this study was verified. For every one standard deviation increase in physical exercise time, there is a corresponding increase of 0.191 standard deviations in adolescents’ prosocial behavior. Both physical health and mental health variables were significant at the 1% level, with a 0.12 standard deviation increase in adolescents’ prosocial behavior for every point increase in physical health, and a 0.082 standard deviation increase in adolescents’ prosocial behavior for every one point increase in mental health. This suggests that physical exercise may indirectly predict adolescents’ prosocial behavior by influencing physical health and mental health channels, i.e., physical health and mental health play a mediating role in physical exercise’s effects on adolescents’ prosocial behavior. At the level of control variables, gender, only-child status, boarding, parents’ highest education attainment, parents’ expectations, parental relationship, school location, and school sports facilities all predict adolescents’ prosocial behavior at different significant levels, while the rest of the control variables do not have a significant effect on adolescents’ prosocial behavior. Specifically, adolescents who are male, only child and boarding, whose parents have a high highest education level, have high expectations, and have good relationships, and whose schools are located in rural areas and are equipped with good sports facilities have a higher level of participation in prosocial behavior.

### 3.2 Robustness check

The estimation results are reported in Table 3, which is consistent with the control variables chosen in Table 2, and Models 1–4 in Table 3 show the estimation results of physical exercise on adolescents’ prosocial behavior, as well as the estimation results with the inclusion of the mediator variables physical health, mental health, and both, in that order. The results show that the estimated coefficients for the core explanation of physical exercise are all significantly positive at the 1% level, indicating that physical exercise does enhance adolescents’ prosocial behavior. In addition, the estimated coefficients of the mediating variables, physical health and mental health, are significantly positive, which is consistent with the results of the baseline regression, proving that the conclusion that physical exercise positively predicts adolescents’ prosocial behavior is robust, and that physical health and mental health may play a mediating role in the influence of physical exercise on adolescents’ prosocial behavior. As a result, the robustness results of the dependent variable method with replacement are consistent with the baseline regression, and Hypothesis 1 of this paper is re-validated.

**Table 3:** Robustness test results: replacing the dependent variable.

Variables	Model 1	Model 2	Model 3	Model 4
Physical exercise	0.085 (7.507)***	0.077 (6.785)***	0.08 (7.105)***	0.075 (6.592)***
Physical health	—	0.081 (7.118)***	—	0.069 (5.91)***
Mental health	—	—	0.065 (5.716)***	0.048 (4.119)***
Control variable	Controlled	Controlled	Controlled	Controlled
Simple size (*n*)	7,605	7,605	7,605	7,605
Constant term	8.923 (0.261)***	8.26 (0.276)***	8.281 (0.284)***	7.881 (0.291)***
*R* ^2^	0.065	0.071	0.069	0.073

***p < 0.001.

### 3.3 Heterogeneity test

The results of the baseline regression and robustness test indicate that physical exercise significantly promotes adolescents’ prosocial behavior. Related research (Hu and Yu, 2019) concluded that adolescents’ individual and familial characteristics predict their physical exercise, and their prosocial behavior varies due to the influence of these factors. So, is there a significant difference between groups in the degree of effects of physical exercise on adolescents’ prosocial behavior? This section focuses on this question (see Table 4).

**Table 4:** Heterogeneity test results.

Variables	Male	Female	High parental education	Low parental education
Physical exercise	0.184 (11.922)***	0.155 (9.613)***	0.178 (5.812)***	0.172 (14.503)***
Physical health	0.127 (7.977)***	0.113 (6.781)***	0.111 (3.468)***	0.122 (9.924)***
Mental health	0.081 (5.147)***	0.089 (5.268)***	0.164 (5.168)***	0.071 (5.828)***
Control variable	Controlled	Controlled	Controlled	Controlled
Simple size	3,679	3,926	6,641	964
Constant term	7.602 (0.336)***	8.515 (0.300)***	8.653 (0.909)***	8.379 (0.243)***
*R* ^2^	0.121	0.101	0.141	0.112

***p < 0.001.

First, physical exercise has a significant positive effect on prosocial behavior for both male and female adolescents, and both are significant at the 1% level. The key estimated coefficient is 0.184 for the male group and 0.155 for the female group, i.e., physical exercise shows stronger effects on the prosocial behavior of male adolescents than that of females.

Second, physical exercise has a significant positive effect on the prosocial behavior of adolescents with high or low parental education, with a key estimated coefficient of 0.178 for adolescents with high parental education, which is significant at the 1% level, and 0.172 for adolescents with low parental education, which is significant at the 1% level, i.e., physical exercise has a stronger effect on the prosocial behavior of adolescents with high parental education than that of adolescents with low parental education.

### 3.4 Mediating role of physical health and mental health

**Figure 2:**
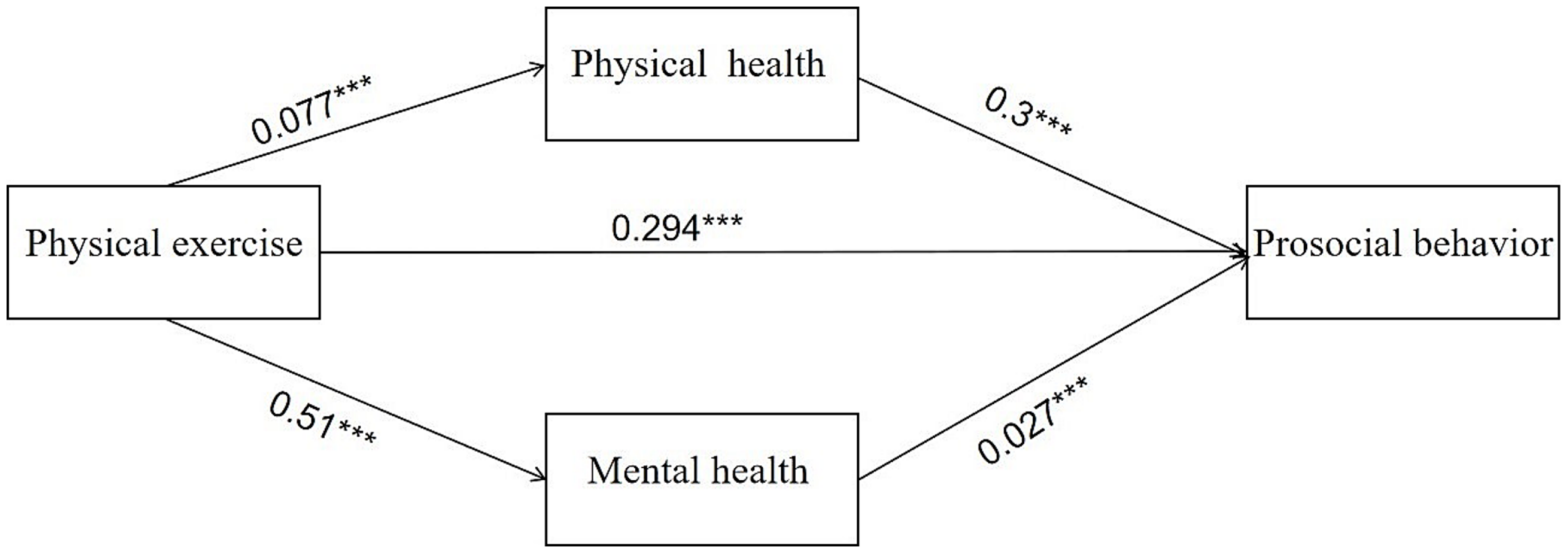
Multiple mediation model of physical and mental health in the relationship between physical exercise and prosocial behavior (path coefficient representation).

In the OLS regression, Model 4 is the estimation result without adding physical health and mental health, and the estimated coefficient (*β*1) of the independent variable physical exercise is 0.191, and with the addition of physical health, mental health, or both, β is 0.177, 0.183, and 0.173, respectively, which are all decreasing to varying degrees but all significant at the 1% level, i.e., physical exercise influences adolescents’ prosocial behavior, but such influence is weakened after the addition of the two. This suggests that physical health and mental health may be the key factors for physical exercise to influence adolescents’ prosocial behavior. In this study, the mediating effects of physical health and mental health were tested using the bootstrap method with 5,000 repeated samples. Table 5 shows the estimation results of the mediating effects of physical health and mental health based on the bootstrap method, and neither of the two channels contains 0 within the 95% confidence interval, which fully indicates significant mediating effects of physical health and mental health in the effects of physical exercise on adolescents’ prosocial behavior. Therefore, Hypotheses 2 and 3 of this study were verified. The total effect of physical exercise on the effects of adolescents’ prosocial behavior is 0.33, of which the direct effect of physical exercise is 0.294, accounting for 89.09%, the mediating effect of physical health is 0.023, accounting for 6.97%, and the mediating effect of mental health is 0.014, accounting for 4.24%. It can be concluded that the effects of physical exercise on adolescents’ prosocial behavior are dominated by the direct effect, and that the mediating effect of physical health is twice as much as that of mental health. The multiple mediation model shown in Figure 2 validates the theoretical model of Figure 1.

**Table 5:** Bootstrap test.

Type of effect	Effect value	Boot standard error	95% confidence interval	Relative effect (%)
Direct effects	0.294	0.017	[0.261, 0.327]	89.09%
Mediating effects of physical health	0.023	0.003	[0.017, 0.030]	6.97%
Mediating effects of mental health	0.014	0.003	[0.009, 0.019]	4.24%
Total effect	0.330	0.017	[0.298, 0.364]	100%

Relative effect (%) represents the proportion of each effect (direct or mediating) relative to the total effect.

In addition, this study additionally used the stepwise method to test the mediating effects of physical health and mental health in the effects of physical exercise on adolescents’ prosocial behavior (Table 6). The coefficient of physical exercise in Model 1 is 0.191 > 0 and significant at 1% level, which represents the existence of the total effect; the coefficient of physical health in Model 2 is 0.097 > 0 and significant at 1% level, and the coefficient of mental health in Model 3 is 0.077 > 0 and significant at 1% level, which represents the fact that physical exercise can significantly improve the physical and mental health of adolescents; the coefficient of prosocial behavior in Model 4 is 0.157 > 0 and significant at 1% level. The coefficient of mental health in Model 5 is 0.133 > 0 and is significant at 1% level, which represents that physical health and mental health can significantly predict adolescents’ prosocial behavior; the coefficient of physical exercise in Model 6 is 0.173 > 0 and is significant at 1% level, which represents the existence of the direct effect of the impact of physical exercise on the adolescents’ prosocial behavior, the coefficient of physical health is 0.12 and the coefficient of mental health is 0.082, which are significant at 1% level, representing the existence of the mediating effect of physical health and mental health. Therefore, Hypotheses 2 and 3 of this study were again validated.

**Table 6:** Step-by-step test.

Variables	Model 1	Model 2	Model 3	Model 4	Model 5	Model 6
	Prosocial behavior	Physical health	Mental health	Prosocial behavior	Prosocial behavior	Prosocial behavior
Physical exercise	0.191 (17.156)***	0.097 (7.664)***	0.077 (6.058)***	—	—	0.173 (15.688)***
Physical health	—	—	—	0.157 (12.484)***	—	0.12 (10.525)***
Mental health	—	—	—	—	0.133 (10.556)***	0.082 (7.245)***
Control variable	Controlled	Controlled	Controlled	Controlled	Controlled	Controlled
*R* ^2^	0.098	0.052	0.047	0.079	0.073	0.122

***p < 0.001.

## 4 Discussion

First, the results of this study indicate a positive association between physical exercise and adolescents’ prosocial behavior, which is consistent with findings from prior research (Hui et al., 2022; Konukman et al., 2022). However, these two studies only collected part of the data in Henan Province and Yangzhou City, and it remains to be verified whether the findings can be extrapolated to other areas (especially rural areas). Moreover, the existing studies have a strong focus on junior high school and college students in the post-COVID-19 period, respectively, and are less relevant to most student groups in normal social environments. A controlled trial by O’Donnell also confirmed the effects of physical exercise on the prosocial behavior of children and adolescents, finding that physical exercise improved their prosocial behavior (O’Donnell and Barber, 2020). The present study advances existing research by providing the first large-scale national evidence in China, analyzing 7,605 adolescents from 112 schools to examine the association between physical exercise and prosocial behavior. By comparing the mediating roles of physical health and mental health in this relationship, our findings offer new insights into how these factors may relate to prosocial behavior among adolescents. This study distinguishes itself from prior research through its national scope and comparative analysis of mediators, which have not been extensively explored in the Chinese context. These results highlight the potential importance of physical exercise for adolescent development and may inform strategies for schools and parents to encourage such activities.

Secondly, the heterogeneity analysis found that the effect of physical exercise on adolescents’ prosocial behavior varied by gender, only child status, boarding, parents’ highest education attainment, parents’ expectations, parental relationship, school location, and school sports facilities. In-depth discussion of the role of physical exercise on the promotion of prosocial behavior of male adolescents is higher than that of females, and previous research has confirmed that the role of parental involvement in promoting the development of prosocial behavior and inhibiting the development of problematic behavior of students has a stronger effect in male students (Wang et al., 2014). In combination with the relevant literature, it may be concluded that adolescents are in a period of rapid physical form development, and that males are more muscular and more inclined to exercise than females due to their physiological characteristics, and are more willing to spend time in exercising (Hu and Yu, 2019). Physical exercise has a more significant effect on the enhancement of prosocial behavior of adolescents with highly educated parents; the family is the starting point for the moral development of children (Li et al., 2024), and more educated parents better understand the significance of their children’s development, which will affect their participation in physical exercise (Tianxiao et al., 2024) and their prosocial behavior (Wang et al., 2014). Therefore, schools and families need to pay extra attention to female adolescents’ participation in physical exercise and create a quality family moral environment and role models.

Finally, mediating effects analysis showed that physical exercise influences adolescents’ prosocial behavior by improving physical health and mental health. Regular and professional exercise with sufficient intensity is beneficial to physical and mental health. Junior high school students are in a period of rapid psychosomatic development, which, coupled with heavy study loads, can lead to the development of physical problems such as obesity (Zhu et al., 2019) and myopia (Li et al., 2017) and psychological problems such as anxiety and depression (Gao et al., 2025). In terms of physical health, previous studies have also concluded that the higher the level of subjective health, the higher the tendency of prosocial behavior, especially among left-behind children (Perc et al., 2012). This may be due to the fact that a physically inactive lifestyle may alter human prosocial behavior by impairing adaptive prosocial decision making in response to social factors through altering functional brain connectivity and inter-brain synchronization (Ishihara et al., 2024). In terms of mental health, based on the theory of positive emotion expansion, physical exercise is effective in positively influencing prosocial behavior by reducing ego depletion and enhancing positive emotions (Yujiang, 2015). This study also concluded that the mediating effect of physical health was twice as large as the mediating effect of mental health in the effects of physical exercise on adolescents’ prosocial behavior, which complements the longstanding research perspective of the academic community that has been overly focused on mental health, and highlights the importance of the role of physical health in the association between physical exercise and prosocial behavior. Therefore, it is possible to enhance adolescents’ sports participation and exercise duration by improving the scientific nature of school physical education curricula and rationally arranging sports programs, so as to strengthen their physical functions, make physical and mental pleasure, and thus promote their prosocial level and overall healthy development.

This study has several limitations that should be noted. First, prosocial behavior is influenced by a multitude of factors, and the mechanisms underlying its interaction with different contexts (e.g., school and family) require further investigation. Future research could employ longitudinal or experimental designs to more rigorously examine the causal relationships between physical exercise and adolescents’ prosocial behavior. Additionally, exploring the dose–response relationship (i.e., the effects of varying intensities or durations of exercise) would provide deeper insights into this association.

## 5 Conclusion

Using data from the 2014–2015 China Education Panel Survey (CEPS), this study employed linear regression analysis and bootstrap testing to examine the relationship between physical exercise and adolescents’ prosocial behavior, along with potential underlying factors. The main findings are as follows: (1) Overall, physical exercise shows a positive association with adolescents’ prosocial behavior, suggesting that increased physical exercise correlates with higher levels of prosocial behavior. (2) Heterogeneity analysis reveals a stronger association among male adolescents than among their female counterparts. Additionally, the relationship between physical exercise and prosocial behavior is more pronounced among adolescents with higher parental education levels. (3) Regarding potential pathways, both physical health and mental health appear to play roles in linking physical exercise to prosocial behavior, with physical health showing approximately twice the mediating association as mental health.

## Data Availability

The datasets presented in this study can be found in online repositories. The names of the repository/repositories and accession number(s) can be found in the article/supplementary material.
